# Genetic dissection of human ABCE1 in yeast reveals separable requirements for ribosome recycling and suppression of aberrant reinitiation

**DOI:** 10.1002/2211-5463.70337

**Published:** 2026-08-31

**Authors:** Eriko Nakata, Yuxin Li, Kei Endo, Koichi Ito

**Affiliations:** ^1^ Laboratory of Molecular Genetics, Department of Computational Biology and Medical Sciences, Graduate School of Frontier Sciences The University of Tokyo Kashiwa Japan

**Keywords:** ABCE1, ribosome recycling, Rli1, *Saccharomyces cerevisiae*, species‐specificity, translational integrity

## Abstract

The ATP‐binding cassette E1 (ABCE1) protein is an essential factor for ribosome recycling, splitting post‐termination ribosomes into subunits for subsequent rounds of translation. Despite high conservation, human ABCE1 (hABCE1) fails to functionally complement the depletion of its essential yeast homolog, Rli1. In this study, we leveraged this species‐specificity to dissect the functional architecture of ABCE1. Through analysis of yeast–human chimeric proteins, we identified the N‐terminal nucleotide‐binding domain (NBD1) as the primary determinant of this incompatibility. To further investigate, we isolated multiple hABCE1 point mutants (revertants) that successfully restored yeast growth. We then developed a novel dual‐luciferase reporter assay to quantify aberrant translation reinitiation in the 3′UTR, an event recognized as a direct consequence of ABCE1 deficiency. Notably, while the revertant mutants rescued yeast viability, they failed to suppress aberrant reinitiation, exhibiting levels equivalent to the nonfunctional hABCE1. This genetic uncoupling of viability from the suppression of reinitiation suggests that the canonical ribosome recycling function required for cell growth and the role in preventing aberrant reinitiation have distinct functional thresholds or are genetically separable aspects of ABCE1 activity.

## Graphical abstract

Human ABCE1 cannot functionally replace its yeast ortholog. Yeast–human chimera analysis identified NBD1 as a major interspecies barrier. Genetic screening yielded hABCE1 revertants that rescue yeast viability but fail to suppress aberrant translation reinitiation in the 3′ UTR. This illustrates the genetic uncoupling of ABCE1's roles: low‐threshold 80S splitting (viability) and high‐threshold 40S retention (translational integrity).

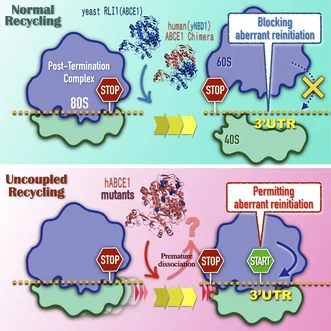

**Abbreviations**
ABCE1: ATP‐binding cassette E1
FLuc: Firefly luciferase
hABCE1: human ABCE1
NBD: Nucleotide‐Binding Domain
NLuc: NanoLuc
Tet: tetracycline
yABCE1: yeast ABCE1 (Rli1)

In eukaryotes, the ATP‐binding cassette protein E1 (ABCE1), known as Rli1 in *Saccharomyces cerevisiae*, plays an essential role in the final stage of protein synthesis: ribosome recycling. ABCE1 utilizes ATP hydrolysis to dissociate the post‐termination 80S ribosome into 40S and 60S subunits, making them available for new rounds of translation [1, 2, 3]. Depletion of ABCE1 in yeast has been shown by ribosome profiling to cause 80S ribosomes to accumulate at stop codons and increased occupancy in the downstream 3′ untranslated region (3′UTR). Consequently, these unrecycled ribosomes can then scan into the 3′UTR and reinitiate translation from noncanonical start sites, generating cryptic peptides [4].

Understanding the mechanisms governing such noncanonical translation events is critical, as ribosome profiling has revealed a ‘translatome’ of far greater complexity than previously anticipated [5]. However, the precise regulation of reinitiation and its physiological impact remain largely enigmatic. Because defects in ribosome recycling and the subsequent accumulation of aberrant proteins are closely linked to various inherited diseases and pathological outcomes [6], it is crucial to elucidate the roles of key quality control factors that ensure the accuracy of protein synthesis.

The function of ABCE1 is critically dependent on a sophisticated network of intra‐molecular allosteric communication—such as that between the NBD1, NBD2, and Hinge domains—and intermolecular interactions with co‐evolved partners. For instance, its ATPase activity for ribosome recycling is triggered by a specific interaction between its NBD1 domain and the ribosomal stalk [7]. Furthermore, ABCE1 remains a core component of the 40S subunit long after splitting, integrating into the 43S pre‐initiation complex (PIC). In this state, its conformation and activity are modulated by other factors, such as the N terminus of eIF3j in humans, as demonstrated in native PICs [8]. This reliance on a network of co‐evolved, species‐specific interactions likely underlies its complex functions.

Despite the high degree of sequence and functional conservation of translation factors, human ABCE1 (hABCE1) is unable to functionally substitute for yeast Rli1, a rare case of species‐specificity. This functional incompatibility provides a unique opportunity to probe the structure–function relationships within this essential protein [9]. In this study, we used this yeast–human chimerism as a genetic tool to identify the domains critical for ABCE1 function. Furthermore, we developed a convenient dual‐luciferase reporter assay, based on the reinitiation phenomenon [4], to quantitatively assess ABCE1's role in suppressing aberrant reinitiation. This approach allows us to investigate the relationship between its canonical role in ribosome recycling (as assayed by cell viability) and its function in maintaining translational integrity (by preventing aberrant reinitiation), which has previously been difficult to dissect from qualitative growth assays or large‐scale ribosome profiling.

## Materials and methods

### Yeast strains and media

The *Saccharomyces cerevisiae* strains used in this study are listed in Table 1. The conditional knockout strain S19‐H06 (tet‐aptamer‐OFF *RLI1*) was constructed based on the BY4727 [10] background as previously described [11]. Yeast cells were grown in YPD (1% yeast extract, 2% peptone, 2% dextrose) or synthetic complete (SC) medium containing the appropriate amino acid dropout mix (ForMedium: Formedium Ltd., UK). To repress the expression of the chromosomal *RLI1* gene in the S19‐H06 strain, tetracycline (Tet) was added to the medium at a final concentration of 100 μm. To establish a stable reporter strain, the dual‐luciferase reporter cassette was integrated into the S19‐H06 genome using the pAUR101TEF integrating vector, a derivative of pAUR101 (Takara Bio: Takara Bio Inc., Kusatsu, Shiga, Japan). Transformants were selected on YPD plates containing 1.0 μg·mL^−1^ Aureobasidin A (AbA), and successful integration at the *AUR1* locus was confirmed, yielding the S19‐H06AUR strain.

**Table 1:** *S. cerevisiae* strains in this study. The table lists the isolated mutations, including the amino acid position (Mutation Number), the original amino acid, the mutated amino acid, the structural feature of the mutation site, and the frequency of isolation from the screen detailed in Fig. 3.

Name	Genotype	References
BY4727	MATα his3Δ200 leu2Δ0 met15Δ0 trp1Δ63 ura3Δ0	[10]
S19‐H06	tetO_7_‐TATA‐RLI1 in BY4727 background	[9]
S19‐H06AUR	S19‐H06, aur1::pAUR101TEF‐Luc‐3′CWP2‐NLuc	This study

### Plasmid construction

The yeast–human chimeric ABCE1 constructs (Chimeras 1–5) and the reverse chimera (Chimera 6) were generated by overlap extension PCR using KOD‐Plus‐Neo DNA Polymerase (Toyobo, Co. Ltd., Japan). Plasmids p415ADH‐yABCE1 and p415ADH‐hABCE1 were used as templates for the initial domain amplifications. Primers were designed to create ~ 20–30 bp overlapping sequences at the junction points between the yeast and human segments. To facilitate reproducibility, the primers are systematically designated according to their binding junctions. For internal domain exchanges (e.g., Chimera 2), which require two junction points, the forward and reverse primer pairs for the 5′ and 3′ junctions are denoted as F1/R1 and F2/R2, respectively. Conversely, terminal domain exchanges (Chimeras 1 and 5) require only a single junction primer pair, denoted simply as F/R. Briefly, individual domains were amplified in a first round of PCR, and the purified fragments were subsequently assembled and amplified into full‐length chimeric open reading frames (ORFs) in a second round of PCR. For this final full‐length amplification, the universal outer primers, ADHprom and CYCter, were utilized for all chimeras. The specific primer combinations used for each fragment and the final assembly are detailed in Table 2. Plasmids used in this study are listed in Table 3. To eliminate template DNA, the assembled PCR products were treated with DpnI prior to purification. The resulting chimeric ORFs were digested with PstI and SalI and cloned into the corresponding sites of the p415CYC1 vector [12]. Importantly, all functional assays (growth complementation and translation reinitiation) were strictly performed using untagged ABCE1 variants to preclude any potential steric hindrance or interference from the tag on ABCE1's highly coordinated interactions with the ribosome. For the chimeric proteins, these variants were expressed from the relatively weak CYC1 promoter to approximate physiological expression levels. However, for the isolation and growth complementation of hABCE1 revertants, the stronger GPD promoter was utilized, as the CYC1 promoter proved too weak for effective mutant screening. To independently assess steady‐state protein expression in yeast, the respective ABCE1 variant sequences lacking their stop codons were PCR‐amplified and inserted into the PstI and SalI sites of p415GPD‐3xFLAG. This modified p415GPD vector contains a C‐terminal 3xFLAG tag sequence inserted between the SalI and XhoI sites, allowing for in‐frame fusion of the target genes. Because the CYC1 promoter yielded insufficient signals for reliable immunodetection of the tagged variants, the stronger GPD promoter was specifically utilized to facilitate western blot analysis. All constructs were verified by DNA sequencing.

**Table 2:** Primers used in this study. The table lists the names, sequences, and descriptions of the oligonucleotide primers used for plasmid construction and mutagenesis. In the specific primer names, ‘h’ or ‘hA’ denotes human ABCE1, and ‘y’ or ‘yR’ denotes yeast ABCE1 (Rli1). F1/R1 and F2/R2 indicate the forward and reverse primers for the 5′ and 3′ junctions, respectively. Primers were designed based on the sequence alignment and domain boundaries defined in Table 5 and Fig. 1. BamH1‐RICWP2‐Nluc‐F and BamH1‐CWP2‐Nluc‐R1 were also designed as alternative assembly primers but the stepwise extension method described above was primarily utilized. A standard universal primer (e.g., M13R) was used as the reverse primer to amplify the downstream region of pCK9 including the BglII site.

Name	Sequence (5′ to 3′)	Usage
V‐F	TCGTCATTGTTCTCGTTCCC	Vector (forward)
V‐R	GGGACCTAGACTTCAGGTTGTC	Vector (reverse)
C1‐H‐R	GGTGAGTTACATGGGCTTCTAAGTTGCTTGGTAGATTGACAATTG	Chimera 1 (human)
C1‐Y‐F	TTAGAAGCCCATGTAACTCACC	Chimera 1 (yeast)
C2‐Y‐R	AAATTAGTTGGCAAATTGATAATTTG	Chimera 2 (yeast segment 1)
C2‐H‐F	CAAATTATCAATTTGCCAACTAATTTGGAAAAAGAAACCACACAT	Chimera 2 (human)
C2‐H‐R	GCCATCCAAAAAAATGTTTATG	Chimera 2 (human)
C2‐Y‐F	CATAAACATTTTTTTGGATGGCCATATTCCTGCTGAAAACCTG	Chimera 2 (yeast segment 2)
C3‐Y‐R	ACCGTCCAAGAATATGTTGATAC	Chimera 3 (yeast segment 1)
C3‐H‐F	ATCAACATATTCTTGGACGGTTATGTTCCAACAGAAAACTTGAGA	Chimera 3 (human)
C3‐H‐R	GACTAGCAGAGTCATTCTGCAATTCATTTGCTGTCTCAGCC	Chimera 3 (human)
C3‐Y‐F	TTGCAGAATGACTCTGCTAGTC	Chimera 3 (yeast segment 2)
C4‐Y‐R	GTCTTCGGTAGCATCAGCTATT	Chimera 4 (yeast segment 1)
C4‐H‐F	GCTGATGCTACCGAAGACGAAGAAGTTAAAAAGATGTGTATGTAT	Chimera 4 (human)
C4‐H‐R	TCCCTTCTGAAGGTGACATTAAGCTGAGACAAAAATTTATTCATG	Chimera 4 (human)
C4‐Y‐F	AATGTCACCTTCAGAAGGGAT	Chimera 4 (yeast segment 2)
C5‐Y‐R	ATCTCTTCTGAATGTAATTTCCAAATTCTTCAAAAATCTGTTACA	Chimera 5 (yeast)
C5‐H‐F	GAAATTACATTCAGAAGAGATCCAA	Chimera 5 (human)
C6‐H‐R	GTTGCTTGGTAGATTGACAATTG	Chimera 6 (human)
C6‐Y‐F	CAATTGTCAATCTACCAAGCAACTTAGAAGCCCATGTAACTCACC	Chimera 6 (yeast)
C6‐Y‐R	ATCTCAAGTTTTCTGTTGGAACATAACCGTCCAAGAATATGTTGATAC	Chimera 6 (yeast)
C6‐H‐F	TATGTTCCAACAGAAAACTTGAGAT	Chimera 6 (human)
Sal1‐C‐h	ATGCGTCGACATCATCCAAGAAAAAGTAGTTTCC	3xFLAG C‐term fusion
Sal1‐C‐y	ATGCGTCGACAATACCGGTGTTATCCAAGAAA	3xFLAG C‐term fusion
BamH1‐CWP2‐Nluc‐	ATGCGGATCCCTAGCTGCTGCTGCTATGTTGTTATAAGAAATCTCT GATTTTTTATAATATCTATATGGCTTTTTTCAAA	2nd forward primer
CWP2‐Nluc‐ΔATG	GATTTTTTATAATATCTATATGGCTTTTTTCAAAATTTTCGGTTTT ACTAGGTCCGTTTTCACTTTGGAAGA	1st forward primer

**Table 3:** Vectors and plasmids in this study. This table lists the 22 oligonucleotide primers used to generate chimeric constructs (Chimeras 1–6) via overlap extension PCR. Systematic naming (e.g., C2‐H‐F) indicates the chimera number, the species origin of the template (H for human *ABCE1*, Y for yeast *RLI1*), and the primer orientation. All ABCE1 variants are inserted in the PstI‐SalI site of the expression vectors.

Name	Description	Reference/source
p415CYC	CEN/ARS vector with CYC1 promoter and LEU2 marker	[12]
p415ADH	CEN/ARS vector with ADH1 promoter and LEU2 marker	[12]
p415TEF	CEN/ARS vector with TEF1 promoter and LEU2 marker	[12]
p415GPD	CEN/ARS vector with GPD1 promoter and LEU2 marker	[12]
p416CYC	CEN/ARS vector with CYC1 promoter and URA3 marker	[12]
p415ADH‐yABCE1	p415ADH harboring yeast RLI1 (yABCE1) ORF, PCR template plasmid for chimeras	This study
p415ADH‐hABCE1	p415ADH harboring human ABCE1 (hABCE1) ORF, PCR template plasmid for chimeras	This study
p415CYC‐yABCE1	p415CYC harboring yeast RLI1 (yABCE1) ORF	This study
p415CYC‐hABCE1	p415CYC harboring human ABCE1 (hABCE1) ORF	This study
p415CYC‐ABCE1 chimeras	p415CYC harboring ABCE1 chimera ORFs for spot assay	This study
p415GPD‐hABCE1 (revertants)	hABCE1 with point mutations (T111I, F331L, I374V, L392I, A393T, T543I, M555V/T, or L580P)	This study
p416CYC‐yABCE1	p416CYC harboring yeast RLI1 (yABCE1) ORF	This study
p416CYC‐hABCE1	p416CYC harboring human ABCE1 (hABCE1) ORF	This study
p415TEF‐Luc2	Luc2 gene w/o stop codon fused to BglII site is inserted in the p415TEF vector	Laboratory stock
p415TEF‐Luc‐3′CWP2‐NLuc	Reinitiation Reporter vector: Luc‐*CWP2* 3′UTR‐NLuc dual‐luciferase reporter (as in Fig. 5A) in the p415TEF vector	This study
pAUR101	Chromosomal integrating *E. coli*–yeast shuttle vector with an Aureobasidin A‐resistance marker (*AUR1‐C*)	Takara Shuzo
pAUR101TEF	pAUR101‐derived expression vector constructed by inserting the *SphI*–*SalI ADH1* terminator fragment from pGAD424 into pAUR101, followed by the insertion of the *TEF1* promoter‐MCS region from p415TEF via the *SacI*–*SalI* sites	This study
pAUR101TEF‐Luc‐3′CWP2‐NLuc	Reinitiation reporter cassette subcloned from p415TEF‐Luc‐3′*CWP2*‐NLuc into the SpeI‐SalI sites of pAUR101TEF	This study
p415GPD3xFLAG	p415GPD derivative containing a C‐terminal 3xFLAG tag sequence inserted between SalI and XhoI sites, allowing in‐frame fusion of target genes via the SalI site	Laboratory stock
p415GPD3xFLAG‐ABCE1 variants	The ABCE1 variant sequences (lacking stop codons) were PCR‐amplified from the respective expression vectors listed above using a common forward primer and a specific reverse primer (Sal1‐C‐h for the human‐type or Sal1‐C‐y for the yeast‐type C‐terminus). The amplified fragments were inserted into the PstI and SalI sites of the p415GPD‐3Flag vector	This study

To construct the dual‐luciferase reinitiation reporter (p415TEF‐Luc‐3′CWP2‐NLuc), the specific CWP2 reinitiation window [4] was fused to the NanoLuc (NLuc) sequence utilizing a stepwise overlapping PCR strategy (Fig. 5A, left). The NLuc sequence was amplified from the pCK9 plasmid (Promega Corporation, Madison, WI, USA). To strictly evaluate translation reinitiation and prevent leaky expression from the reporter itself, the first two native ATG start codons of NLuc were initially excluded via PCR, and the sequence was designed to fuse directly starting from the subsequent TCC (Serine) codon. The CWP2 sequence, encompassing its native stop codon and the reinitiation window, was then appended to the 5′ end of this ΔATG‐NLuc fragment in subsequent overlapping PCR steps. The resulting PCR product, containing a 5′ BamHI site, the CWP2 stop codon and reinitiation window, the NLuc(ΔATG) sequence, and a 3′ BglII site, was digested with BamHI and BglII. This fragment was subsequently ligated into the unique BglII site of the p415TEF‐Luc vector, directly downstream of the luc2 gene. The ligation of BamHI and BglII compatible cohesive ends generated a hybrid AGATCC site, establishing an in‐frame fusion that terminates precisely at the +1 frame stop codon of the CWP2 sequence, accurately recreating the reinitiation window geometry (Fig. 5A, right). For genomic integration, this entire dual‐luciferase reporter cassette was readily excised from p415TEF‐Luc‐3′*CWP2*‐NLuc utilizing the unique SpeI and SalI sites (Fig. 5A) and subsequently subcloned into the corresponding sites of the pAUR101TEF integrating vector to generate pAUR101TEF‐Luc‐3′CWP2‐NLuc. Prior to yeast transformation, this plasmid was linearized with the appropriate restriction enzyme, StuI, within the *AUR1‐C* gene to direct homologous recombination.

### Yeast transformation and growth complementation assay

Yeast transformation was performed using the standard method. For growth complementation assays (spot assays), transformants were cultured overnight in selective liquid medium (e.g., SC‐Leu). The cultures were then adjusted to an OD_600_ of 0.5, serially diluted (10‐fold), and 5 μL of each dilution was spotted onto SC‐Leu plates with or without 100 μm tetracycline. Plates were incubated at 30 °C for 4–8 days and photographed; specific incubation periods for each experiment are noted in the corresponding figure legends.

### Isolation of hABCE1 revertant mutants

To isolate hABCE1 mutants that complement yeast growth, the hABCE1 coding sequence in the p415GPD vector was randomly mutagenized by error‐prone PCR. The resulting pool of PCR fragments and a linearized p415GPD vector were cotransformed into the S19‐H06 strain. Transformants were selected on SC‐Leu plates containing 100 μm tetracycline (Fig. 3A). Plasmids were recovered from a large number of colonies that initially exhibited restored growth. To eliminate potential false positives, the isolated plasmids were amplified in *Escherichia coli* and subsequently re‐introduced into the naive S19‐H06 yeast strain. Only the plasmids that consistently conferred a robust growth‐complementation phenotype upon this second round of transformation were retained for further analyses. The entire ORF of the hABCE1 gene from these confirmed candidatures was sequenced to identify the point mutations. For simplicity of functional dissection, we prioritized candidates with a single point mutation. Variants containing multiple amino acid substitutions were omitted from subsequent analysis to preclude secondary or synergistic effects that could complicate the interpretation of individual residue functions.

### Dual‐luciferase reinitiation assay

Translation reinitiation was quantified using a dual‐luciferase reporter plasmid, which was constructed based on previously described findings [4] using a p415TEF‐based vector under the control of the *TEF1* promoter (*TEF1*p). The reporter contains a codon‐optimized firefly luciferase (*luc2*) gene as the main ORF. The *luc2* ORF is designed to terminate at a stop codon in a different reading frame (+1 frame) relative to the downstream reporter. Following the stop codon, the 3′UTR sequence from the *CWP2* gene, containing a known reinitiation window, was inserted. To specifically monitor reinitiation, a NanoLuc (NLuc) gene lacking its own initiation codon (ATG‐NLuc) was fused in‐frame to the reinitiation site within this window.

For the assay validation (Fig. 5), yeast strains harboring the reporter plasmid alone were grown on SC‐Leu agar plates (Fig. 5B), whereas strains coharboring the reporter plasmid and the URA3‐marked expression vectors were grown on SC‐Ura, Leu agar plates (Fig. 5C). For the quantitative evaluation of ABCE1 variants (Fig. 6), the S19‐H06AUR strain harboring the integrated reporter and specific LEU2‐marked expression vectors was grown on SC‐Leu agar plates. Subsequently, cells were cultured overnight in 1 mL of the respective selective liquid medium. The next morning, the cultures were diluted to an OD600 of 0.2, and tetracycline was added to induce expression. After approximately 4 h of incubation, the final OD600 was recorded. For the luciferase assay, 64 μL of the culture was mixed with 16 μL of 5× Passive Lysis Buffer (Promega) and incubated with shaking for 10 min. Luminescence was measured using the Nano‐Glo^®^ Dual‐Luciferase^®^ Reporter (NanoDLR™) Assay System (Promega) according to the manufacturer's instructions. Briefly, 80 μL of ONE‐Glo™ EX Reagent was added, and after 10 min of shaking, firefly luciferase (FLuc) activity was measured. Subsequently, 80 μL of NanoDLR™ Stop & Glo^®^ Reagent was added, and after an additional 10 min of shaking, NanoLuc^®^ luciferase (NLuc) activity was measured. The reinitiation efficiency was calculated as the ratio of NLuc to FLuc activity. Experiments were performed in at least three independent biological replicates (*n* = 3) unless otherwise indicated. Values were normalized to the efficiency observed in the presence of wild‐type yABCE1 (RLI1) or as specified in the figure legends.

### SDS/PAGE and Western blot analysis

Total protein extracts were prepared from yeast cultures by mechanical disruption using glass beads (4 m/s for 40 s). Briefly, harvested yeast cells were resuspended in extraction buffer containing 20 mm Tris–HCl (pH 7.6), 50 mm KCl, and 10 mm MgCl_2_. Cells were disrupted with acid‐washed glass beads (425–600 μm) at room temperature for 20 s. The supernatant was collected as the protein sample after subsequent centrifugation steps. Samples were resolved by SDS/PAGE on TGX FastCast Acrylamide gels (Bio‐Rad Laboratories: Hercules, CA, USA) at 200 V for 40 min and transferred to a PVDF membrane using the Trans‐Blot Turbo Transfer System (Bio‐Rad Laboratories). The membrane was blocked with Blocking One (Nacalai Tesque: Kyoto, Japan) for 5 min at room temperature and incubated with primary antibodies: anti‐FLAG (1 : 5000, M2; Sigma‐Aldrich: St. Louis, MO, USA) or anti‐PGK1 (1 : 5000, 22C5D8; Thermo Fisher Scientific: Waltham, MA, USA) for 1 h. After washing, the membrane was incubated with an anti‐mouse IgG, peroxidase‐linked species‐specific whole antibody (from sheep), NA931 (1 : 10 000; GE Healthcare: Chicago, IL, USA) for 30 min. Chemiluminescent signals were developed using ImmunoStar LD (FUJIFILM Wako Pure Chemical: Osaka, Japan) and detected with ImageQuant™ LAS 500 Chemiluminescent Imaging System (Cytiva: Marlborough, MA, USA).

## Results

### Analysis of yeast–human chimeric proteins identifies the NBD1 domain as the locus of species‐specific incompatibility

**Fig. 1:**
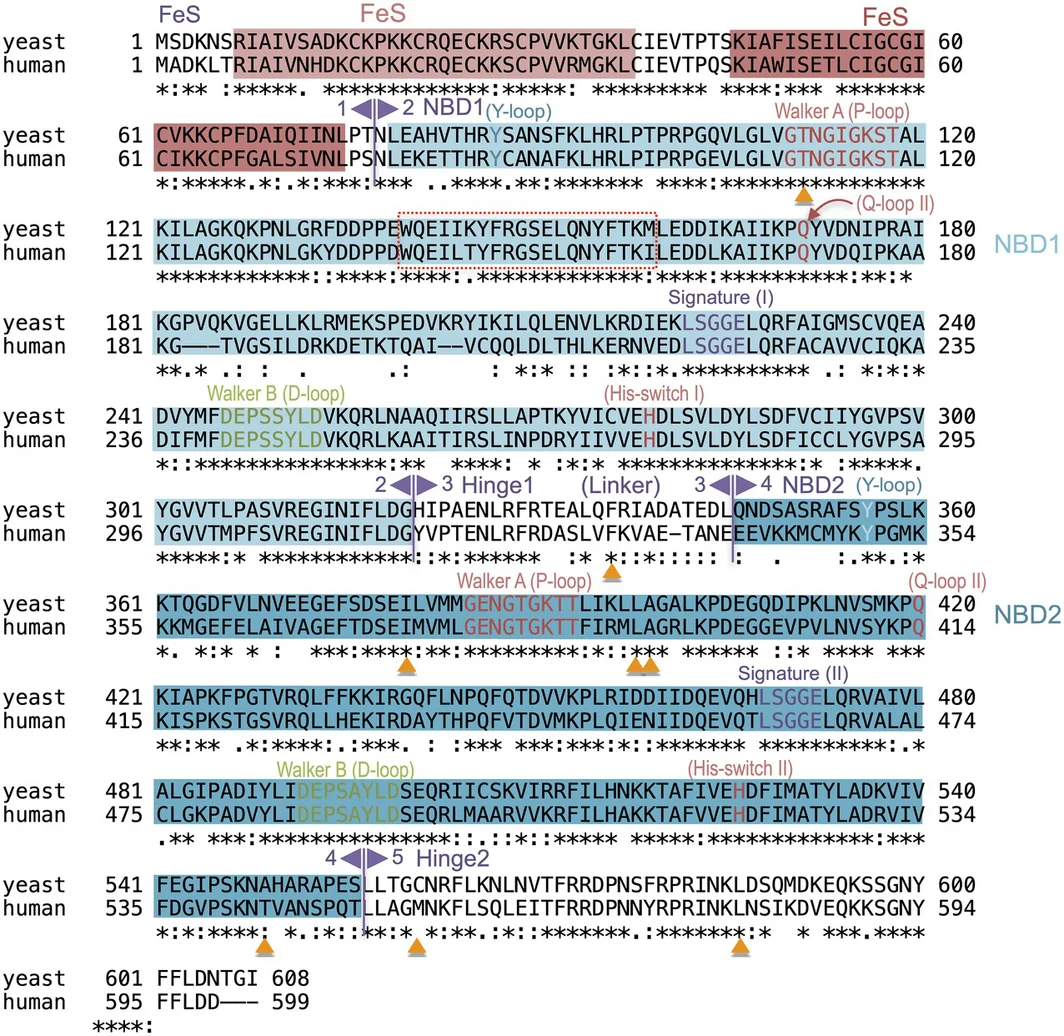
Sequence alignment of *S. cerevisiae* and human ABCE1. The amino acid sequences of yABCE1 (top) and hABCE1 (bottom) were aligned using clustalw. The boundaries of the five domains used for constructing the chimeric proteins (see Fig. 2) are indicated by boxes. Conserved functional motifs, including the Walker A/B motifs, Signature sequences, Q‐loops, and Hinge regions, are labeled along the alignment. The positions of the revertant mutations (Fig. 3 and Table 4) are marked with arrows. yABCE1, yeast ABCE1; hABCE1, human ABCE1.

**Fig. 2:**
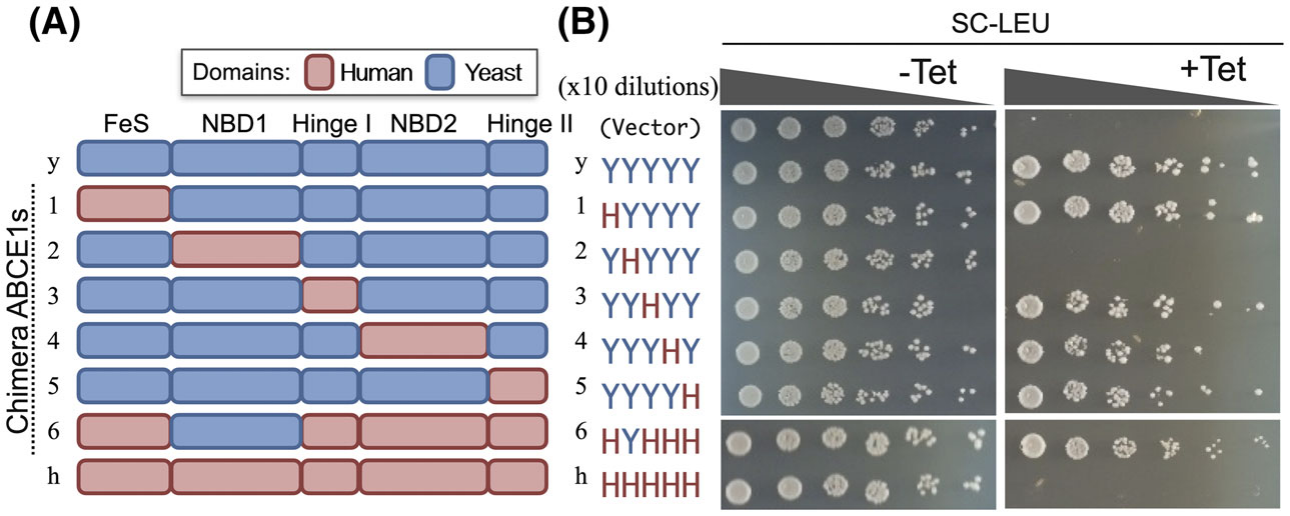
The NBD1 domain of ABCE1 is a key determinant for species‐specific function. (A) Schematic representation of the yeast–human ABCE1 chimeric proteins. The five domains (FeS, NBD1, Hinge I, NBD2, Hinge II) of yeast ABCE1 (yABCE1) were systematically replaced with their human counterparts. The reverse chimera (hABCE1 with yeast NBD1) is also shown. Schematic representation of the domain architecture. Domain lengths are approximate to allow visualization of shorter regions. Exact domain boundaries used for cloning are indicated in Fig. 1 and Table 5. Note that the depicted domain lengths are adjusted for visual clarity and do not strictly reflect their actual sizes. (B) Growth complementation assay of the chimeric constructs. The S19‐H06 strain, in which endogenous *RLI1* expression is repressed by tetracycline (+Tet), was transformed with plasmids expressing the indicated ABCE1 variants. Serial dilutions (×10) of cells were spotted on selective SC‐Leu agar plates with (+Tet) or without (−Tet) tetracycline and incubated at 30 °C. The empty expression vector (Vector) was used as a negative control. Images are representative of three independent experiments with similar results. Plates were incubated at 30 °C for 4 days. ABCE1, ATP‐binding cassette E1; FeS, iron–sulfur cluster; NBD, nucleotide‐binding domain; Tet, tetracycline; SC‐Leu, synthetic complete medium lacking leucine.

To identify the domain responsible for the functional incompatibility of hABCE1 in yeast, we constructed a series of chimeric proteins between yeast Rli1 (yABCE1) and hABCE1. Based on an alignment of the two orthologous proteins (Fig. 1), we systematically replaced each of the five structural domains of yABCE1 (FeS, NBD1, Hinge I, NBD2, and Hinge II) with the corresponding human domain to generate Chimeras 1–5 (Figs 1, 2A and Materials and Methods). We tested the ability of these constructs to support growth in a yeast strain where the endogenous *RLI1* gene is translationally repressed by tetracycline [11]. As shown in Fig. 2B, swapping the FeS, Hinge 1, NBD2, or Hinge 2 domains of yABCE1 with their human counterparts (Chimeras 1, 3, 4, 5) had no significant growth defect. This indicates that these four domains are functionally interchangeable between yeast and humans. In sharp contrast, replacing only the NBD1 of yABCE1 with its human counterpart (Chimera 2) failed to restore yeast growth. Conversely, a ‘reverse chimera’ (Chimera 6), consisting of hABCE1 with the yeast NBD1 domain, successfully restored yeast growth. These results demonstrate that the NBD1 domain is the primary determinant governing the species‐specific incompatibility of hABCE1 in yeast. The precise functional defect conferred by this domain swap, however, remains to be determined and will be explored further in the Discussion section.

### Isolation of point mutations in hABCE1 that restore yeast growth

**Fig. 3:**
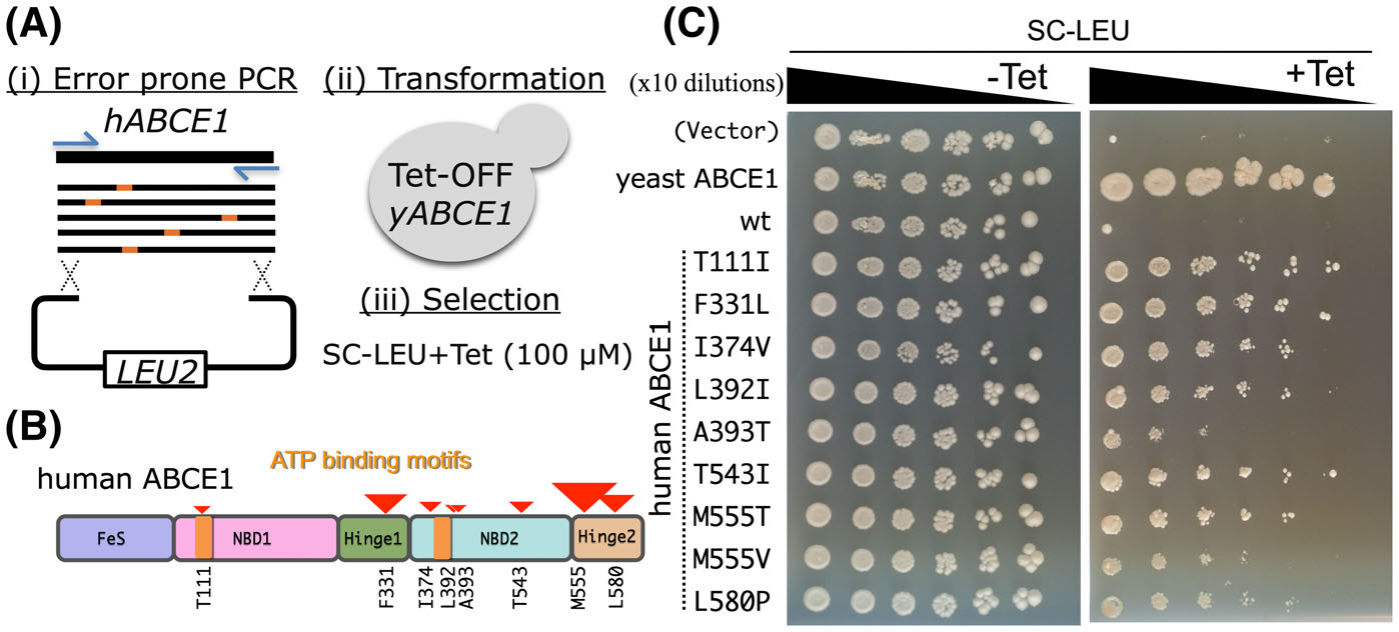
Isolation and characterization of hABCE1 mutants that restore yeast growth. (A) Schematic of the genetic screen to isolate hABCE1 mutants. The screen consists of three main steps. (i) The hABCE1 coding region was amplified from its expression vector under error‐prone PCR conditions using primers that anneal to the promoter and terminator regions. (ii) The resulting pool of mutagenized DNA fragments was co‐transformed into the *RLI1* translationally‐repressible strain along with the p415GPD expression vector, which had been linearized by digestion at the multiple cloning site. The circular plasmids expressing various hABCE1 mutants were then reconstituted *in vivo* via homologous recombination. (iii) Transformants were selected on medium containing tetracycline (100 μm). Plasmids were recovered from viable colonies, the growth‐complementing phenotype was re‐confirmed by a second round of transformation, and the causative mutations were identified by DNA sequencing. (B) Map of isolated hABCE1 mutations on the hABCE1 protein. The frequency of isolation for each mutation site is indicated by the size of the arrow. A detailed list of mutations is provided in Table 4. Regions highlighted in yellow indicate the conserved ATP‐binding motifs (Walker A). Note that the depicted domain lengths are adjusted for visual clarity and do not strictly reflect their actual sizes. (C) Growth complementation assay of the isolated mutations. Yeast cells expressing wild‐type or variant ABCE1 were spotted onto SC‐Leu agar plates as described in Fig. 2B. Images are representative of three independent biological replicates. Plates were incubated at 30 °C for 8 days. hABCE1, human ABCE1; yABCE1, yeast ABCE1; Tet, tetracycline; SC‐Leu, synthetic complete medium lacking leucine.

**Fig. 4:**
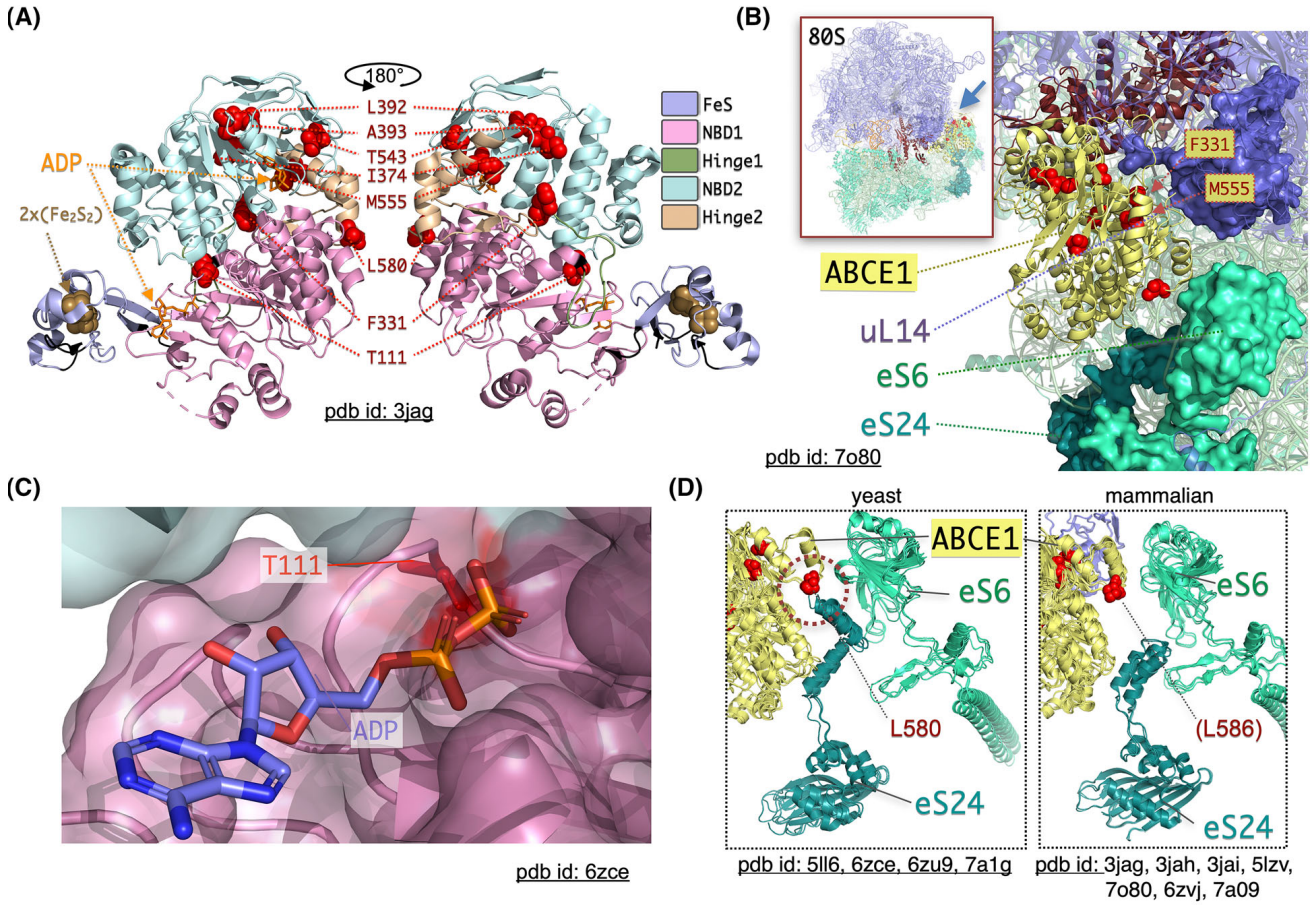
Structural mapping of the isolated hABCE1 revertant mutations. (A) The isolated mutations are mapped onto the three‐dimensional structure of ABCE1 derived from the mammalian ribosomal termination complex (PDB ID: 3JAG). To clearly visualize all mutation sites, the ABCE1 structure is shown in two orientations rotated by 180°. The positions of the substituted amino acid residues (red) and the bound ADP molecules (orange) are indicated by arrows. The five distinct structural domains of ABCE1 are color‐coded according to the key provided in the top right panel. (B) The binding environment of ABCE1 on the 80S ribosome (PDB ID: 7O80). The top‐left inset shows the overall structure of the mammalian 80S ribosome termination complex, with the large and small subunits colored in slate and lime green, respectively. The arrow in the inset indicates the viewing angle for the main panel. In the main panel, ABCE1 is displayed in cartoon representation, while the adjacent ribosomal proteins uL14, eS6, and eS24 are depicted in surface representation. The specific locations of the revertant residues F331 (proximal to uL14) and M555 (at the 40S interface) are explicitly highlighted to illustrate their structural context. eRF1 is visible in the background and colored in chocolate. (C) The location of the T111 residue within the nucleotide‐binding pocket. ABCE1 from the yeast 43S pre‐initiation complex (PDB ID: 6ZCE) is shown in surface representation. The panel highlights the ADP molecule bound to the NBD1 domain and the T111 residue (colored red). The structure illustrates that T111 is not only in contact with the phosphate group of the bound ADP but also occupies a strategic node closely juxtaposed to the NBD2 domain. The color‐coding of the ABCE1 domains is the same as in (A). (D) Structural comparison of the interaction between ABCE1 residue L580 (L586 in yeast) and the C‐terminal domain of eS24. The left and right panels show superimposed structures of ABCE1, eS24, and eS6 from yeast (PDB IDs: 5LL6 [40S post‐splitting complex], and 6ZCE, 6ZU9, and 7A1G [43S/48S pre‐initiation complexes]) and mammalian (PDB IDs: 3JAG, 3JAH, 3JAI, 5LZV, and 7O80 [80S termination pre‐splitting complexes], and 6ZVJ and 7A09 [43S/48S pre‐initiation complexes]) complexes, respectively. The superimpositions illustrate the strong structural conservation within each species group. The dotted circle in the left panel highlights the characteristic contact between mammalian eS24 and ABCE1 L580. In the right panel, the equivalent yeast residue, L586, is indicated for comparison. Molecular graphics and analyses in this figure were performed using the pymol molecular graphics system, Version 3.1.10 (Schrödinger, LLC). PDB, Protein Data Bank; ADP, adenosine diphosphate; PIC, pre‐initiation complex.

Based on our finding that the NBD1 domain dictates species‐specificity, we performed an unbiased genetic screen across the entire hABCE1 sequence to identify interdomain or structural compensatory mutations. Because initial attempts using the weak CYC1 promoter yielded insufficient expression for successful screening, we utilized the stronger GPD promoter (p415GPD vector) for this strategy. We introduced random mutations into the nonfunctional hABCE1 and selected for variants that could restore growth in the ABCE1/RLI1‐translationally repressible tet‐OFF yeast strain on SC‐Leu medium containing 100 μm tetracycline (Fig. 3A; see Materials and Methods for procedural details). As a result, we successfully isolated hABCE1 variants harboring nine distinct point mutations at eight different sites that conferred viability. The locations and isolation frequencies of these mutations are summarized in Fig. 3B and Table 4. While the chimera analysis indicated that the NBD1 domain is the primary determinant of species‐specific incompatibility, the isolated compensatory mutations exhibited a distinct distribution. These mutations were primarily located on the surface of NBD2, in areas close to the ribosomal interfaces, and at key functional sites in other domains that neighbor NBD2. All isolated mutants conferred a partial but significant rescue of the growth defect on SC‐Leu plates containing 100 μm tetracycline, indicating a restoration of, at least, the essential function of ABCE1 required for cell viability (Fig. 3C). To visualize their spatial distribution, we mapped all identified mutation sites onto the ABCE1 structure derived from the mammalian ribosomal termination complex (PDB ID: 3JAG) (Fig. 4A). This specific structure was utilized because both nucleotide‐binding sites and all the mutated residue positions are structurally fully resolved in this state. Furthermore, examination of the structural environment of ABCE1 bound to the 80S ribosome provides a structural context for these mutations. The overall binding topology shows that ABCE1 interacts with both ribosomal subunits, positioning several revertant residues near specific ribosomal proteins (Fig. 4B). Specifically, closer inspection of the twin‐nucleotide‐binding domains reveals that the mutation sites, such as T111I and A393T, are situated in close proximity to the ATP‐binding pockets, flanking the bound nucleotides (Fig. 4C). Moreover, interfacial residues cluster near the contact surfaces with ribosomal proteins uL14, eS6, and eS24 (Fig. 4D) [13, 14]. To ensure a clear genotype–phenotype correlation, we focused strictly on these revertants harboring a single amino acid substitution; the underlying mechanisms of these mutations will be addressed in the Discussion.

**Table 4:** Summary of hABCE1 revertant mutations. Detailed list of compensatory mutations in human *ABCE1* (hABCE1) that restored viability in the *rli1* yeast strain. The table provides the amino acid position in human ABCE1, the specific substitution, the corresponding residue in yeast Rli1, the structural feature of the mutation site (e.g., NBD ATP‐binding pocket or ribosomal interface), and the frequency of isolation from the screen.

Human	Mutated	Yeast	Structural feature of mutation site	Frequency
T111	I	T111	NBD1: Walker A(I)/ATP binding	4
F331	L	F336	Hinge I: Close to uL14 (large subunit) Close to Hinge II of ABCE1	5
I374	V	I380	NBD2	2
L392	I	L398	NBD2: Close to ATP binding pocket	1
A393	T	A399	NBD2: Close to ATP binding pocket	1
T543	I	A549	NBD2: Close to ATP binding pocket	3
M555	V	C561	Hinge II: Close to Large Subunit rRNA	6
M555	T	C561	Hinge II: Close to Large Subunit rRNA	7
L580	P	L586	Hinge II: Close to Large Subunit rRNA, eS24 in Yeast	5

**Table 5:** Domain boundaries for yeast–human ABCE1 chimera construction. Definition of the domain boundaries used for designing the chimeric proteins. Boundaries for the five structural domains (FeS, NBD1, Hinge I, NBD2, and Hinge II) were determined based on the sequence alignment of *Saccharomyces cerevisiae* Rli1 and *Homo sapiens* ABCE1 (shown in Fig. 1). These residue numbers served as the junction points for the overlap extension PCR.

Domain	Yeast RLI1 residues	Human ABCE1 residues	Structural feature
FeS	1–78	1–78	Iron–sulfur cluster domain
NBD1	79–320	79–315	Nucleotide‐binding domain 1
Hinge I	321–344	316–339	Pivot region connecting NBDs
NBD2	345–568	340–562	Nucleotide‐binding domain 2
Hinge II	569–608	563–599	C‐terminal ribosomal anchor

### hABCE1 Revertants rescue yeast growth but fail to suppress aberrant Reinitiation

**Fig. 5:**
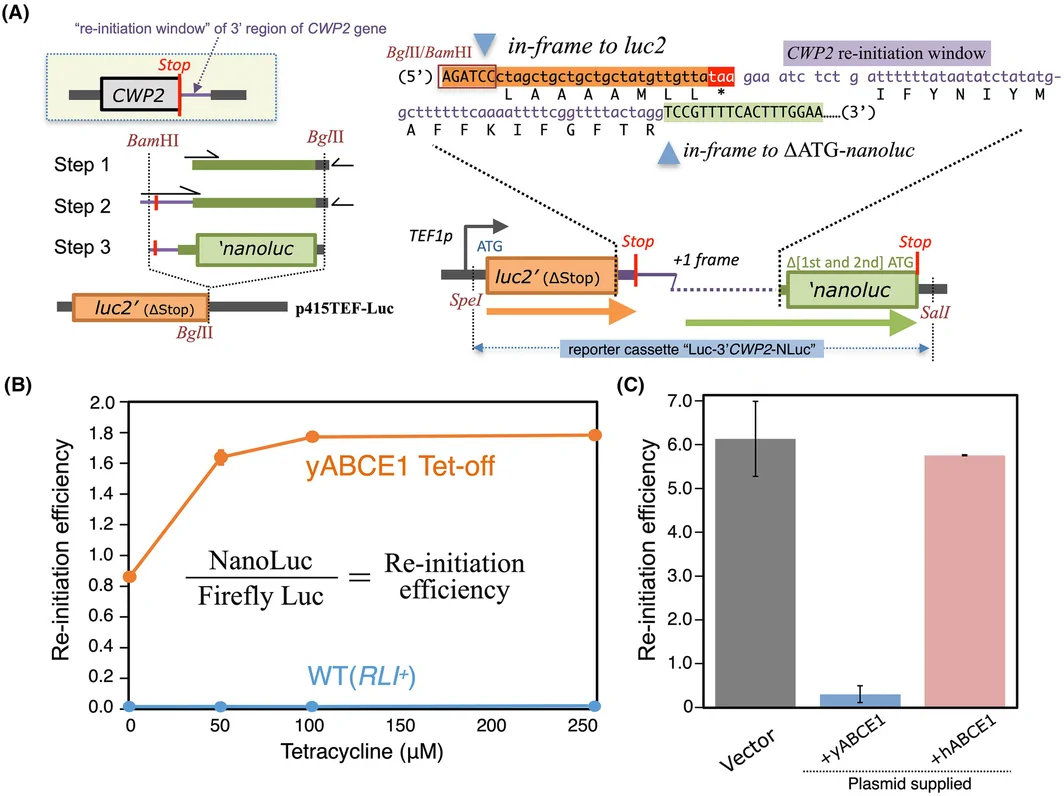
Dual‐luciferase reporter system to quantify translation reinitiation. (A) Construction strategy and sequence design of the dual‐luciferase reporter. (Left) Schematic of the cloning steps. The NanoLuc (NLuc) sequence lacking its first two ATG start codons was amplified (Step 1) and subsequently fused to the yeast *CWP2* sequence (Step 2), which encompasses its native stop codon and reinitiation window, via overlapping PCR. The resulting fragment was digested with BamHI and BglII (Step 3) and inserted into the BglII site downstream of the luc2 gene. (Right) The nucleotide and amino acid sequences around the reinitiation window. The luc2 open reading frame terminates precisely at the +1 frame stop codon of the CWP2 sequence, and translation reinitiation is quantified by the downstream in‐frame ΔATG‐NLuc reporter. (B) Validation of the reporter assay. The formula used to calculate reinitiation efficiency is shown within the graph. The BY4727 (*RLI1* ^+^) and S19‐H06 (yABCE1 Tet‐off) containing the reporter plasmid were cultured (*n* = 2) in SC‐Leu liquid medium with increasing concentrations of tetracycline (0–250 μm) to titrate endogenous yABCE1 levels. Reinitiation efficiency increases as yABCE1 is depleted. (C) Reinitiation efficiency of yeast cells co‐harboring the reporter plasmid and expressing wild‐type yABCE1 or hABCE1 from p416CYC (URA3 marker) in SC‐Leu‐Ura medium. The empty vector was used as a negative control. Data represent the mean ± SD from three independent biological replicates. FLuc, Firefly luciferase; NLuc, NanoLuc; UTR, untranslated region; Tet, tetracycline.

To quantitatively assess the functional consequence of ABCE1 deficiency, we designed a dual‐luciferase reporter assay to measure the rate of translation reinitiation. For this, we focused on the 3′UTR of the yeast *CWP2* gene, where significant reinitiation was previously observed upon ABCE1 depletion [4]. The reporter construct was created by fusing the firefly luciferase (FLuc) ORF, lacking its own stop codon, in‐frame with the *CWP2* 3′UTR, thereby utilizing the natural *CWP2* stop codon. Downstream, a NanoLuc (NLuc) gene was fused in a different reading frame within the reinitiation window identified by the previous study (Fig. 5A). The reinitiation efficiency was then calculated as the ratio of NLuc to FLuc activity (Fig. 5B).

We first validated this assay system. When the reporter was introduced into the parental wild‐type strain, NLuc expression was negligible, as expected. However, in the *RLI1* translationally repressible strain, reducing yABCE1 expression by titrating tetracycline led to a dose‐dependent increase in the reinitiation efficiency (Fig. 5B). Furthermore, this elevated reinitiation was suppressed only when yABCE1 was supplied via a plasmid, but not by an empty vector or hABCE1 (Fig. 5C). Together, these results validate that our assay system accurately measures ABCE1‐dependent reinitiation. It is noteworthy that this repressible strain exhibited a significant basal level of reinitiation even in the absence of tetracycline, likely because the Rli1 protein expression does not reach full wild‐type levels.

**Fig. 6:**
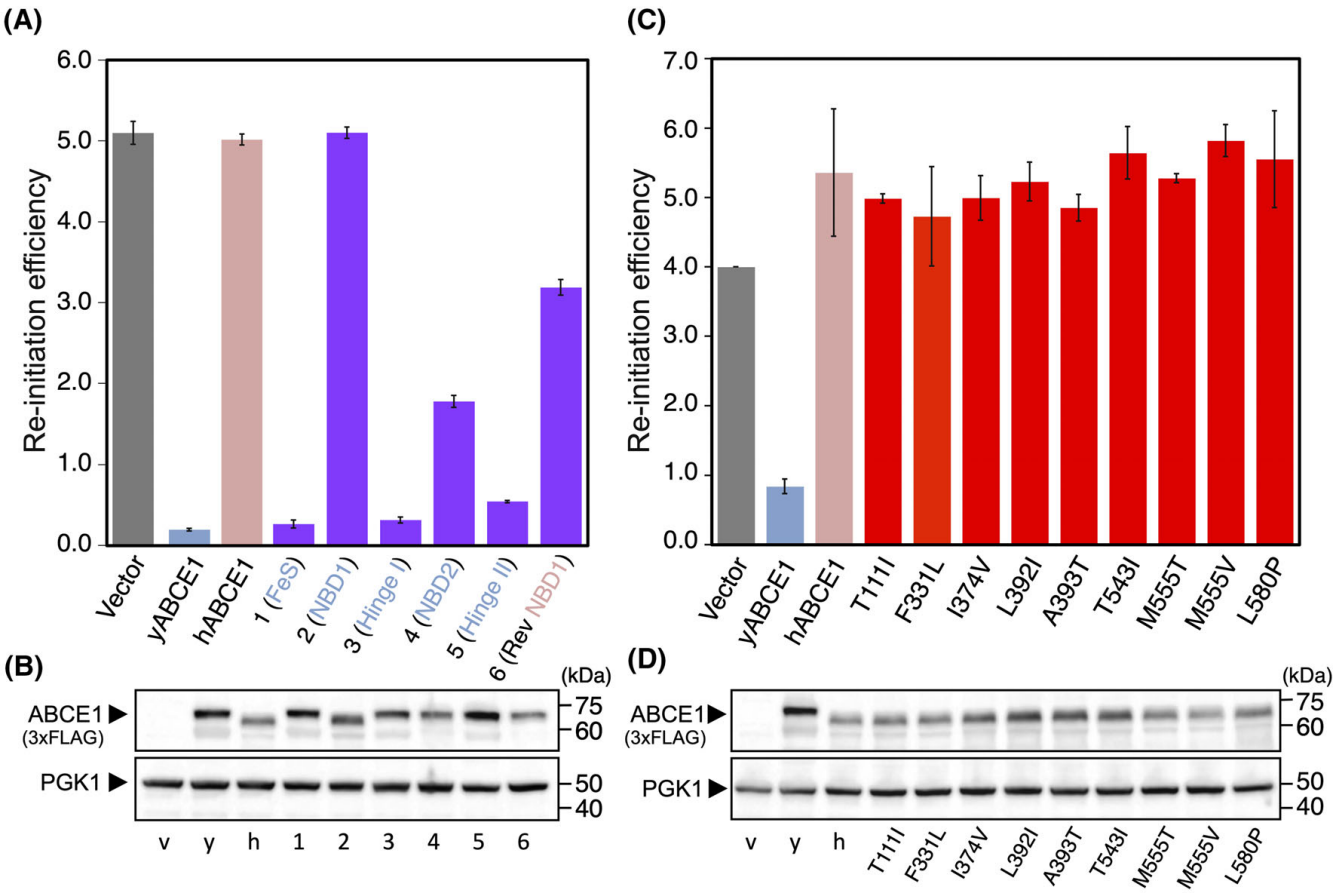
Revertant mutants rescue growth but not reinitiation suppression. (A) Reinitiation efficiency of yeast cells expressing chimeric ABCE1 proteins in S19‐H06AUR cultured in SC‐Leu medium. Data are shown as mean ± SD from three independent experiments. (B) Western blot analysis of C‐terminally 3xFLAG‐tagged chimeric ABCE1 proteins expressed from the p415GPD vector in the S19‐H06 strain. Upper panel: ABCE1 expression levels; lower panel: PGK1 expression levels as a loading control. Lane labels indicate cells transformed with the empty vector (v), wild‐type yABCE1 (y), wild‐type hABCE1 (H), or chimeras 1–6. (C) Reinitiation efficiency of yeast cells expressing hABCE1 revertant mutants in S19‐H06AUR cultured in SC‐Leu medium. Controls include empty vector, wild‐type yABCE1, and wild‐type hABCE1. Data are shown as mean ± SD from three independent experiments. (D) Western blot analysis of C‐terminally 3xFLAG‐tagged hABCE1 revertant mutants expressed from the p415GPD vector in the S19‐H06 strain. The panel layout is identical to (B), showing ABCE1 (upper panel) and PGK1 (lower panel) expression levels, with lane labels v, y, and h defined as in (B) followed by the respective point mutants. Abbreviations: hABCE1, human ABCE1; yABCE1, yeast ABCE1; PGK1, phosphoglycerate kinase 1.

Analysis of the chimeric proteins confirmed that the reinitiation defect is linked to the NBD1 domain (Fig. 6A). To rule out the possibility that the observed functional defects of these chimeras simply resulted from protein instability, we evaluated their steady‐state expression levels. While untagged constructs were utilized for all functional assays to avoid tag‐induced artifacts, parallel western blot analysis using the C‐terminally 3xFLAG‐tagged versions confirmed that all chimeras were detectably and stably expressed, although with some variations in steady‐state levels (e.g., lower expression for Chimera 3 and hABCE1) (Fig. 6B). Importantly, given that Chimera 3 successfully suppresses aberrant reinitiation despite its lower expression, minor fluctuations in protein abundance do not appear to dictate this specific functional outcome.

While the revertant mutations successfully rescued cell viability and allowed for yeast growth (Fig. 3C), they remained profoundly defective in suppressing aberrant reinitiation. All tested revertants failed to suppress aberrant reinitiation, exhibiting efficiencies nearly identical to those of the nonfunctional wild‐type hABCE1 and significantly higher than that of wild‐type yABCE1 (Fig. 6C). Crucially, this failure to suppress reinitiation was not a consequence of reduced protein levels. Western blot analysis of the corresponding 3xFLAG‐tagged variants demonstrated that all hABCE1 revertants maintained steady‐state expression levels equal to or slightly higher than that of wild‐type hABCE1, although inherently lower than that of yABCE1 as previously noted for heterologous expression [9] (Fig. 6D). This comparable expression profile establishes two critical points: First, the failure to suppress reinitiation is not a consequence of reduced protein stability; second, the restoration of cell viability is not due to nonspecific protein accumulation or overexpression. Rather, it underscores that the point mutations qualitatively improved the functional interaction of hABCE1 with the yeast ribosome while preserving the overall structural integrity of the protein.

This result provides clear genetic evidence that the ABCE1 function required for cell viability is distinct and separable from its role in suppressing aberrant reinitiation, at least at the functional threshold required for colony formation.

## Discussion and Conclusions

In this study, we utilized the species‐specificity of ABCE1 as a genetic tool to dissect its multifaceted functions. Our data indicate a functional divergence between the two roles of ABCE1: the hABCE1 point mutants, which we isolated for their ability to partially rescue the lethality of *RLI1* deletion in yeast (Fig. 3C), remain profoundly defective in suppressing aberrant translation reinitiation (Fig. 6C). This genetic uncoupling of cell viability from translational integrity strongly suggests that the essential ribosome recycling function of ABCE1 required for cell growth and its role in suppressing aberrant reinitiation have distinct and separable functional thresholds.

One plausible interpretation of these observations is a ‘partial rescue’ model. The spot assay for growth is largely qualitative, selecting for any mutant that restores ABCE1's ribosome‐splitting activity to a level just sufficient for colony formation. Our mutants clearly meet this low functional threshold. In contrast, our dual‐luciferase assay provides a sensitive, quantitative measure of reinitiation, a direct consequence of inefficient ribosome recycling. The failure of the mutants to suppress reinitiation indicates that their partially restored activity is insufficient to efficiently clear all post‐termination ribosomes from mRNA, demanding a high functional threshold. Thus, we propose that maintaining translational integrity demands a much higher level of ABCE1 activity than cell viability alone.

This then raises the question: What is the primary defect of hABCE1 in yeast? Our chimera analysis indicates that the incompatibility is not a result of minor mismatches across the protein but is governed almost exclusively by the NBD1 domain. Our data show that the FeS, H1, NBD2, and H2 domains are fully interchangeable between species (Fig. 2B). In contrast, swapping the NBD1 domain solely was sufficient to either break (Chimera 2) or restore (Chimera 6) full function—including not only robust growth but also the suppression of aberrant reinitiation (Fig. 6A). This suggests that the NBD1 domain serves as a critical species‐specific interface.

A crucial insight comes from biochemical studies [7], which demonstrated that the ATPase ‘power stroke’ of ABCE1 is allosterically triggered by a direct, physical interaction between its NBD1 domain and the C terminus of the ribosomal P‐stalk protein. We propose that this NBD1‐stalk interaction represents the ‘first species‐specific interface’ for ABCE1 function. The hABCE1‐NBD1 likely fails to properly engage the yeast P‐stalk, leading to a significant impairment of ATPase activation, inefficient ribosome recycling, and thus both lethality and high reinitiation. The NBD1 chimera (Chimera 6) rescues function because it ‘repairs’ this primary interface.

### Structural implications of revertant mutations for co‐evolved ribosomal interfaces

It is noteworthy that no single point mutations were isolated within the NBD1 domain itself. This absence suggests that the sequence divergence of NBD1 between species is too extensive to be functionally rescued by a single amino acid substitution, further supporting the chimera analysis that NBD1 represents the primary species‐specific barrier. Historically, mutational analyses of ABCE1 within homologous systems (e.g., yeast‐in‐yeast) have predominantly yielded loss‐of‐function phenotypes, leading to lethality or severe growth defects, particularly when targeting the conserved NBD Walker motifs or Hinge regions [14, 15]. In contrast, the suppressor mutations isolated in our heterologous yeast–human chimera system function as gain‐of‐function variants that actively overcome species‐specific incompatibilities. The emergence of these compensatory mutations provides intriguing insights into how flexibly ABCE1 can tune its structural dynamics to adapt to its co‐evolved interaction partner, the ribosome.

This bipartite structural mismatch, comprising a primary NBD1‐stalk signaling defect and a 40S anchoring defect, provides a robust framework for interpreting our panel of revertant mutations. How can single point mutations compensate for these complex, species‐specific defects? Our detailed analysis of the revertants (Table 4) provides a compelling answer. The mutations are not randomly distributed; they cluster in distinct functional locations and appear to overcome the species barrier via two different strategies: (1) direct, physical repair of co‐evolved ribosomal interfaces, and (2) allosteric compensation within the ATPase engine itself.

The majority of our isolated mutations (23 of 34 total clones) fall into the first class: Interfacial Compensators. These mutations may alter local flexibilities or conformations to compensate for physical or chemical mismatches between hABCE1 and the yeast ribosome, rather than establishing new direct physical contacts. The most frequent revertant, M555V/T (combined Freq: 13; 6 for M555V and 7 for M555T) at a conserved site, illustrates this mechanism. Located in Hinge II, hM555 aligns with yC561, revealing a significant chemical mismatch at this 40S anchor point (a bulky hydrophobe vs. a small polar/cysteine residue). The M → V (smaller) or M → T (polar, more Cys‐like) substitutions correct this incompatibility, allowing the Hinge II domain to properly dock onto its rRNA/protein partner on the yeast 40S subunit, as noted in Table 4, which might contribute to stabilizing the postsplitting complex and preventing premature dissociation of ABCE1.

Similarly, the F331L mutations (Freq: 5) are in Hinge I, a region that undergoes pivotal rearrangements during the ATPase cycle and, as shown in Table 4, resides proximal to the 60S protein uL14 [13, 15]. The substitution of a large aromatic Phenylalanine (F) with smaller Leucine (I/L) residues may mitigate steric hindrance with the yeast 60S subunit, permitting the NBD closure required for the 80S splitting power stroke by potentially providing the Hinge I region the necessary flexibility to effectively transmit conformational changes from the NBDs.

Furthermore, the L580P mutation (Freq: 5), which also resides in the Hinge II domain, links our data to the ribosomal protein eS24 [8, 14]. This contact represents the ‘second species‐specific interface’ we propose. While previous structural and cross‐linking studies have demonstrated that disrupting the Hinge–ribosome interfaces results in a severe loss of function [15], our isolated mutation paradoxically restores viability. As illustrated in Fig. 4D, a superimposition of representative yeast ABCE1‐ribosome complexes (e.g., PDB IDs: 5LL6, 6ZCE, 6ZU9, and 7A1G) reveals that the extended C‐terminal domain of yeast eS24 lies in close proximity to the Hinge II domain, establishing a direct contact with L586 (the yeast equivalent of mammalian L580). In striking contrast, this interaction remains undetected in currently available human structures, where the C‐terminal helices of eS24 are kinked and folded back, failing to reach the hL580 residue. Notably, while this contact is consistently visible across multiple yeast structures regardless of the translation stage, its absence in human models may not reflect a genuine lack of this interaction *in vivo*. Given the evolutionary conservation of these regions, this structural discrepancy might rather be attributed to the weak and transient nature of the interaction, rendering it highly susceptible to disruption during biochemical isolation or structural analysis. Crucially, the fact that the L580P mutation was isolated as a functional revertant demonstrates that this transient contact is universally critical for the functional mode of ABCE1, regardless of the species. The L → P substitution potentially introduces a localized structural bend or rigidity. Rather than disrupting the fold, this alteration may stabilize the Hinge II loop into a conformation that effectively mimics or enhances this transient yet essential contact with yeast eS24, thereby operating as a gain‐of‐function compensator.

### Possible mechanisms of functional restoration through allosteric mutations

While the interfacial mutations discussed above likely restore essential physical contacts, a second, less frequent class of mutations (11 of 34 events) acts as Allosteric Compensators. These mutations cluster in or near the NBD ATP‐binding sites (T111I, I374V, L392I, A393T, T543I). Instead of fixing the physical interfaces, they appear to promote enzymatic activity, thereby circumventing the requirement for ribosomal activation signals.

The T111I mutation (Freq: 4) exemplifies this allosteric mechanism. As depicted in Fig. 4C using the structure of the yeast 43S pre‐initiation complex (PDB ID: 6ZCE), T111 is uniquely positioned within the NBD1 Walker A (P‐loop) motif. Previous studies have established that mutations in the Walker motifs typically abolish ATP binding or hydrolysis, leading to a complete loss of function and cell death [15, 16]. However, the isolation of the T111I substitution as a gain‐of‐function suppressor strongly implies that the ATP hydrolysis cycle of human ABCE1's NBD1 is inherently uncoupled or not optimally tuned when bound to the yeast ribosome. It not only directly contacts the phosphate group of the bound nucleotide in the NBD1 pocket but is also situated at a crucial interface adjacent to NBD2. This strategic location allows T111 to intimately link the functional cycles of both NBDs.

The isolation of the T111I suppressor mutation strongly implies that the ATP hydrolysis cycle of human ABCE1's NBD1 is not optimally tuned when bound to the yeast ribosome. The substitution of a polar threonine with a hydrophobic isoleucine could theoretically modulate the hydrolysis efficiency in one of two distinct directions to overcome this incompatibility. For instance, it might intrinsically enhance the basal hydrolysis rate, effectively bypassing the requirement for the missing ribosomal signal. Alternatively, by decreasing the hydrolysis rate, the mutation could prolong the lifespan of the ATP‐bound closed conformation [16], ensuring sufficient time for the mechanical separation of the ribosomal subunits to occur.

This mechanistic interpretation also resolves the apparent paradox between our chimera and point mutation analyses. While replacing human NBD1 with its yeast counterpart yields a fully functional protein (Fig. 2B), the isolated point mutations that afford partial rescue predominantly map to NBD2 or the interdomain interfaces. How can mutations in NBD2 compensate for a primary defect residing in NBD1?

Because ABCE1 operates as a strictly coupled twin‐ATPase [16], mutations in NBD2 might allosterically rescue NBD1 defects. In an allosteric bypass scenario, NBD2 mutations might stabilize the ATP‐occluded closed conformation of the entire cassette, driving NBD1 into its active state without the yeast ribosomal signal. Alternatively, in a kinetic coupling model, NBD2 mutations might decelerate the overall ATPase cycle, extending the dwell time of the closed conformation and providing NBD1 with a sufficient temporal window for ribosome splitting. Importantly, while these NBD2‐driven patches provide enough basal recycling activity for cell survival, they fall short of restoring the finely tuned kinetics required for suppressing aberrant reinitiation.

The remaining NBD2 mutations (I374V, L392I, and A393T) stabilize the NBD2‐ATP ‘closed’ state. These mutations are predicted to enhance hydrophobic packing within the core or near the ATP‐binding pocket. Consistent with principles of ABC transporter dynamics [17], these substitutions to more hydrophobic residues likely shift the conformational equilibrium toward the dimerized state, functionally mimicking the fully ATP‐bound form even under suboptimal allosteric stimulation. This conformation is known to be the stable ‘hybrid state’ in native 43S PICs [8] and allosterically promotes NBD1 activity. These mutations compensate for the lack of proper allosteric signals from *both* the NBD1‐stalk and Hinge‐eS24 interfaces. Interestingly, the T543I mutation is located at the boundary between NBD2 and the Hinge II domain. Rather than solely affecting the NBD2 core, this substitution may act as an allosteric transducer, facilitating the transmission of conformational signals from the yeast ribosome‐Hinge II interface directly into the NBD2 ATP‐binding pocket.

This two‐class suppression model accounts for the observed differences between the constructs. The low‐threshold function (viability) can be partially rescued by *either* strategy: (1) physically repairing the key ribosomal anchor points (M555V/T, L580P, F331L) or (2) allosterically ‘hot‐wiring’ the NBDs (T111I, etc.). However, neither strategy restores the system to wild‐type. The high‐threshold function (suppressing reinitiation) likely requires the complete, co‐evolved allosteric network to be intact: a perfectly timed NBD1‐stalk activation *and* a stable Hinge‐eS24 anchor on the 43S PIC. The isolated revertants likely represent partial evolutionary adaptations rather than full functional restorations. They restore just enough function for the cell to survive, but not enough to execute the high‐fidelity function, revealing a deep functional dependence on species‐specific co‐evolution.

From a mechanistic perspective, this phenotypic uncoupling clearly reflects the distinct physical constraints of the two sequentially coupled phases of ABCE1 operation: an initial catalytic phase for 80S splitting and a subsequent structural retention phase on the 40S subunit [8, 13, 16]. The compensatory mutations provide sufficient mechanical force to overcome the kinetic barrier for the catalytic splitting, ensuring cell viability. However, stable retention on the 40S subunit requires an extensive and highly specific thermodynamic binding interface [4, 13]. Because the revertants cannot fully repair this underlying structural mismatch, their low binding affinity likely results in premature dissociation from the 40S subunit, thereby permitting unwanted reinitiation. Thus, the requirements for dynamic ribosome splitting and static ribosome retention are functionally and genetically separable.

In conclusion, our work provides the first genetic evidence that the distinct roles of ABCE1—supporting viability and suppressing aberrant reinitiation—can be genetically uncoupled, suggesting distinct functional thresholds for each role. By combining yeast genetics with insights from genome‐wide translational analyses, we have established a versatile experimental approach, including a novel and validated reporter assay and a series of partial gain‐of‐function alleles that uncouple viability from the suppression of aberrant reinitiation. This platform has allowed us to propose a ‘two‐interface’ model for ABCE1's co‐evolution with the ribosome, highlighting a previously unappreciated functional divergence in its regulation at the 43S PIC level (eS24 in yeast vs. eIF3j in humans). This system will serve as a valuable tool for future mechanistic studies to dissect the complex, allosteric coordination of ribosome recycling and translational integrity.

## Conflict of interest

The authors declare no conflict of interest.

## Author contributions

KI and KE supervised projects. EN planned experiments. EN and YL performed experiments and analyzed the data. KI and EN wrote the paper.

## Data Availability

The data that support the findings of this study are available from the corresponding author upon reasonable request.
